# Online survey on barriers and drivers to flu vaccination among staff at a German university hospital during the Covid-19 pandemic 2022 (flu-vaccination motive study)

**DOI:** 10.3205/dgkh000519

**Published:** 2024-12-16

**Authors:** Annika Schmidt-Bandelin, Thomas Kohlmann, Alexander Ruback, Uwe Reuter, Axel Kramer

**Affiliations:** 1 Executive Department Company Medical Service, University Medicine Greifswald, Germany; 2 Institute of Hygiene and Environmental Medicine, University Medicine Greifswald, Germany; 3 Institute for Community Medicine, Section Methods in Community Medicine, University Medicine Greifswald, Germany; 4 Division Organization & Development of Healthcare of the Medical Board of Director, University Medicine Greifswald, Germany; 5 Executive Board of the University Medicine Greifswald, Germany

**Keywords:** flu vaccination, barriers, drivers, online survey, health care workers, trainees; students, occupational medical services, influence COVID-19 pandemic, flu-vaccination motive study

## Abstract

**Introduction:**

The success of flu vaccination depends primarily on the willingness of health care workers (HCWs) to be vaccinated. To identify barriers and drivers to vaccination, an online survey among employees and students of a university hospital was performed to develop a local strategy to increase the vaccination willingness in line with the WHO recommendation.

**Method:**

A cross-sectional, anonymous, self-administered online survey was performed among HCWs, other staff, trainees and students of the Greifswald University Hospital between 17.02.2022 and 17.03.2022.

**Results:**

Of 4,709 online questionnaires, 1,515 were answered (response rate 32.2%). 45.3% stated that they were vaccinated annually, 33.4% irregularly and 7.7% had been vaccinated once. 13.6% had never been vaccinated. The proportion of non-vaccinated was highest among trainees and students (25.4%).

5.7% of those vaccinated said the willingness to be vaccinated has decreased because of the pandemic, and 12.1% felt encouraged. 5.8% of those vaccinated at least once did not want to be vaccinated in the future; 14.8% were undecided. The reasons for non-vaccination were dominated by perception of low assumed risk of infection (62.1%), followed by doubts about vaccine efficacy (22.8%) and fear of side effects (13.8%).

**Conclusions:**

Because a timely reminder of vaccination was essential for 16.7% of staff, the occupational medical service will intensify the annual vaccination campaign within the hospital. Additionally, staff will be provided with a brief sheet on the importance of flu vaccination every year before the flu season with the option of personal advice from the occupational medical service. In a lecture, medical students are informed about the benefits of the flu vaccination.

## Zusammenfassung

**Einleitung:**

Der Erfolg der Grippeschutzimpfung hängt maßgeblich von der Bereitschaft der Beschäftigten im Gesundheitswesen ab, sich impfen zu lassen. Um die Hindernisse und Triebkräfte für die Impfung zu ermitteln, wurde eine Online-Befragung unter Mitarbeitern und Studenten eines Universitätsklinikums durchgeführt, um eine lokale Strategie zur Erhöhung der Impfbereitschaft im Einklang mit der WHO-Empfehlung zu entwickeln.

**Methode:**

Zwischen dem 17.02.2022 und dem 17.03.2022 wurde eine anonymisierte, selbstverwaltete Online-Querschnittsbefragung unter Beschäftigten und sonstigen Mitarbeitern des Universitätsklinikums, Auszubildenden und Studenten durchgeführt.

**Ergebnisse:**

Von 4.709 online Fragebögen wurden 1.515 beantwortet (Teilnehmerrate 32,2). Hiervor gaben 45,3% an, jährlich geimpft, 33,4% unregelmäßig geimpft und 7,7% einmal geimpft worden zu sein. 13,6% waren noch nie geimpft. Der Anteil der Nichtgeimpften war bei Auszubildenden und Studenten am höchsten (25,4%).

5,7% der Geimpften gaben an, dass die Bereitschaft, sich impfen zu lassen, aufgrund der Pandemie abgenommen hat, 12,1% fühlten sich ermutigt. 5,8% der mindestens einmal Geimpften wollten sich in Zukunft nicht mehr impfen lassen; 14,8% waren unentschlossen. Bei den Gründen für die Nichtimpfung dominierte die Wahrnehmung eines geringen Infektionsrisikos (62,1%), gefolgt von Zweifeln an der Wirksamkeit des Impfstoffs (22,8%) und der Angst vor Nebenwirkungen (13,8%).

**Schlussfolgerungen:**

Da eine rechtzeitige Erinnerung an die Impfung für 16,7% der Mitarbeiter wichtig war, wird der arbeitsmedizinische Dienst die jährliche Impfkampagne innerhalb des Krankenhauses intensivieren. Zusätzlich wird dem Personal jedes Jahr vor der Grippesaison ein kurzes Informationsblatt über die Bedeutung der Grippeimpfung ausgehändigt, verbunden mit dem Angebot der persönlichen Beratung durch den arbeitsmedizinischen Dienst. Studierende der Humanmedizin werden in einer Vorlesung auf den Nutzen der Grippeschutzimpfung hingewiesen.

## Introduction

Although vaccination remains the most effective method of reducing seasonal influenza morbidity and mortality, particularly in at-risk groups [1], only around half of healthcare workers in Europe let themselves be vaccinated [2]. Through vaccination, the risk of influenza in the population during seasons when the circulating flu viruses closely match those used for vaccine production can be reduced by 31% to 62.5% [3], [4], [5], [6], ICU admissions due to severe courses of influenza can be reduced by 26% [7] or 82% [8], and the mortality rate by 31% [7]. Moreover, flu vaccination is associated with lower complication rates in patients with diabetes [9], or heart and chronic lung diseases [10], [11]. Furthemore, vaccination was associated with reducing absenteeism rates [12]. For healthcare workers (HCWs), there is an ethical aspect to vaccination to protect patients and reduce nosocomial flu infections [13], [14], [15]. 

The success of flu vaccination depends primarily on the availability of vaccination for HCWs and their willingness to be vaccinated [16]. In terms of willingness, it is important to understand advantages of being vaccinated to reduce motivational barriers and increase vaccination willingness. Besides the generally known barriers to vaccination (such as the 5 myths about the flu and/or flu vaccine: influenza is not serious, the vaccine can induce flu and severe side effects, vaccination is not effective, gravidity is a contraindiction [17], [18]) and the frequently mentioned reasons for not getting a vaccination (e.g., low risk of disease, lack of time or opportunity, lack of vaccination promotion, negative attitudes toward the flu vaccine, or belief that there are other methods of preventing flu [19]), it is important to understand local barriers in order to develop locally adapted strategies to increase vaccine uptake [20]. Therefore, this study invesitaged the main reasons for non-utilization of the vaccination among the staff at the Greifswald University Hospital (GUH). Because the COVID-19 pandemic has influenced the willingness be vaccinated against the flu, with rates in Germany increasing in the 2020/21 season, but decreasing in the 2021/22 season [21] while internationally increasing [22], the survey assessed whether an influence of the COVID-19 pandemic on the willingness to get vaccinated against influenza is detectable at the GUH.

## Materials and methods

A cross-sectional, anonymous, self-administered online survey was performed among HCWs, other staff, trainees and students at the GUH between 17.02.2022 and 17.03.2022. The participants were informed about the purpose of the flu-vaccination motive study and the voluntary, confidential, and anonymous nature of the study. The questionnaire (see Attachment 1) was developed on the basis of the first and last authors’ own considerations and tested for suitability in a pre-test (n=20). Besides socio-demographic data, the following questions with options were asked:

- Received influenza vaccinations in your life (never, once, twice or more, annually),
- reasons for non-vaccination (doubts about protective effect, fear, low personal infection risk, existing contraindications, other),
- side effects after vaccination in the past,
- influence of COVID-19 pandemic on willingness to be vaccinated against the flu (yes, no); if yes, increased (to prevent influenza) or decreased willingness to get vaccinated (e.g., skepticism about necessity of influenza vaccination),
- motivation for flu vaccination (multiple answers: self-protection, protection of others [e.g., family, patients, etc.]), previous influenza infection, occupational indication, pregnancy, risk of COVID-19 infection, pre-existing illness,
- reasons to get the flu vaccination (own initiative, reminder by employer/supervisor, recommendation of general practitioner, public reporting in press, radio, television and internet),
- who administered the vaccine (occupational medical service at GUH, general practitioner, another doctor [gynecologist, public health office, etc.]),
- satisfaction with the flu vaccination,
- willingness to be vaccinated against the flu in the future (yes, no).

To ensure anonymity, processing activities and contact processing were developed in cooperation with GUH’s quality management. After approval of the list of processing activities by the GUH's data protection department and the GUH’s Works Council, all questions were entered and stored in the EVASYS survey system.

On 17.02.2022, all employees and students were asked via e-mail to fill out the questionnaire online, using the link to EVASYS. After two weeks, a reminder e-mail was sent. On 17.03.2022, the questionnaire collection ended by deactivating the link. 

The questionnaire was made available to employees and students via an internal website generated by the survey system. This meant that the survey was only available online in a lot-based procedure. A lottery-based survey is freely accessible to all persons who receive the link to the survey. Data analysis was performed using the SPSS statistical package, version 25. Group comparisons were conducted, with differences between groups analyzed using Pearson’s Chi-squared tests. 

## Results

### Baseline data

1,515 employees participated in the flu-vaccination motive study, which is a response rate of 32.2%. Regarding gender distribution, the sample reflects the situation at the GUH. Compared to the overall age structure of employees structure at the GUH in the sample, the age group 21 to 30 years was slightly underrepresented and the other age groups were slightly overrepresented. Regarding the type of service, proportionally more doctors and scientific as well administrative personal and proportionally fewer nursing and therapeutic personal as well as trainees and students took part (Table 1 (Tab. 1)).

Over three-quarters of vaccinated respondents were vaccinated by the occupational medicine service of the GUH (Table 2 (Tab. 2)). The vaccinated respondents reported fewer complications than after vaccination outside the GUH (p=0.039; Table 2 (Tab. 2)).

### Uptake of flu vaccination

Respondents replied as follows: 45.3% are vaccinated annually, 33.4% irregularly, 7.7% had only received one flu vaccination, and 13.6% had never been vaccinated against influenza (Table 3 (Tab. 3)).

The difference in vaccine coverage between the various types of service/occupational areas was significant (p<0.001). The proportion of previously unvaccinated individuals was highest among trainees and students (25.4%), followed by administrative employees (19.7%). The lowest rate of previously unvaccinated individuals was found in the group of doctors and scientists (7.4%; Table 4 (Tab. 4)). 

Vaccination uptake increased with age (p<0.001). In the age group under 21 years, 25.7% had not been vaccinated. Among those over 60 years, only 8.3% reported not having received vaccination (Table 5 (Tab. 5)). 

### Intention to get vaccination

79.4% of respondents want to be vaccinated in the future, 5.8% not and 14.8% were undecided. Also, if side effects occurred, 68% intended to get vaccinated in the future. Intention to be vaccinated was higher among those who did not report to have experienced side effects (Table 6 (Tab. 6)). 

Future willingness to be vaccinated against influenza varied significantly (p<0.001) by type of occupation. It was highest among doctors and scientific services. Most undecided respondents were in the group of trainees and students (Table 7 (Tab. 7)). 

Readiness for future vaccinations was higher given an occupational indication (p=0.002; Table 8 (Tab. 8)).

Of those who already had an influenza infection in the past, 90.4% want to be vaccinated against influenza again in the future. Respondents who had already experienced an influenza infection were more likely to be willing to get vaccinated against influenza in the future (p=0.014; Table 9 (Tab. 9)).

### Reasons for vaccination

Among participants who had been vaccinated at least once (n=1.309), self-protection (75.7%) and protection of others (51.2%) were by far the most frequently mentioned reasons for vaccination uptake. 30.6% were motivated for occupational reasons. The least mentioned reasons for flu vaccination were pregnancy (2.2%), pre-existing health conditions (6.6%), previous influenza infection (6.9%), and the risk of contracting a SARS-CoV-2 infection (7.1%; Table 10 (Tab. 10)).

When the motives are stratified by occupation, self-protection dominated (between 81.8% and 91%) and was the primary motive among technicians, followed by administration staff and doctors/scientifics. Protection of others dominated among doctors/scientists, followed by technical service staff (Table 11 (Tab. 11)). 

A large proportion of vaccinated individuals (83.3%) opted for vaccination on their own initiative. 22.4% took advantage of the employer's offer (Table 12 (Tab. 12)). 

Pre-existing illness increased the willingness for future flu vaccination (p=0.042; Table 13 (Tab. 13)). Individuals with chronic health conditions were more likely to be vaccinated than those without. 

### Reasons for non-vaccination

Among those who had never been vaccinated against influenza, the perception of a low risk to themselves of infection (62.1%) dominated, followed by doubts about the protective effect (22.8%) and fear of side effects (13.8%) (Table 14 (Tab. 14)). There was no difference in reasons for not being vaccinated against the flu among the different staff groups and trainees/students. 

### Willingness to be vaccinated in the future

About future willingness for vaccination, 79.4% of respondents who had previously been vaccinated at least once against influenza, wanted to be vaccinated again, 5.8% did not want vaccination and 14.8% were undecided.

In the group vaccinated for self-protection, 85.4% intended to continue being vaccinated against influenza. Among those not vaccinated for self-protection, the group of undecided individuals was particularly large at 40.4% (p<0.001; Table 15 (Tab. 15)). Among those who were vaccinated against influenza for reasons of other people’s protection, 66.1% were willing to be vaccinated against influenza again in the future (p<0.001; Table 15 (Tab. 15)).

90.4% of those who had previously experienced an influenza infection intended to get vaccinated against influenza again (p=0.014; Table 16 (Tab. 16)).

### Influence of the COVID-19 pandemic on uptake of flu vaccination

12.1% of the vaccinated respondents stated that the pandemic had positively influenced their decision to get their flu shot, and 5.7% replied negatively. The majority of respondents felt unaffected by the pandemic in terms of their attitude toward vaccinations by the pandemic. With increasing age more respondents stated they were not influenced by the COVID-19 pandemic. Especially the younger age groups reported an increase in vaccination willingness due to the COVID-19 pandemic (p=0.029; Table 17 (Tab. 17)). The influence was least among the nursing and therapeutic service (p=0.003; Table 18 (Tab. 18)). There was no influence of gender and organizational unit on the uptake of flu vaccination.

## Discussion

### Material and method

A pre-test was used to ensure that the questions were clear and comprehensible. Suggestive items were avoided when designing the questions. For some questions, multiple answers were possible.

The lot-based survey is suitable when an opinion is to be obtained from an unknown group of participants.

Accessing the internet link or entering the password does not allow identification of the response in the survey; nor is the function of the participation overview activated in the survey. However, the password-based method allows multiple participations in the survey. This function was not considered as confounding factor in the evaluation of the survey, as it is unlikely that someone would answer the questionnaire multiple times.

### Results

#### Rate of vaccination

The vaccination rates among HCWs in the GUH clearly exceed the average vaccination rate of HCWs in Europe [2] as well as in Germany. In the nationwide online survey conducted by the Robert Koch Institute Berlin, the vaccination rate in the saison 2019/20 was 55% [22], and in 2022/23 [23] as well as 2023/24, it was 58% [24]. One reason may be that 99.1% of the employees were aware of the vaccination offer and 93.4% had stated that the clinic had informed them on-site about the offer. This underscores the importance of comprehensive information to all staff about the vaccination offer. Otherwise, the fact that around 1/10 of HCWs and 1/4 of trainees and students were unvaccinated underscores the ethical necessity of intensifying persuasion in these groups. Following the previous annual campaigns for vaccination by the medical director and the occupational medical service, the subordinate levels of responsibility will be included in the campaign in the future. This includes the clinic and institute directors, nursing management, the staff unit for infection control and, in each medical department, the link physician and link nurse for infection control. Especially the role model of clinic directors can increase vaccination readiness.

The fact that the age groups under 30 and over 40 years were underrepresented in the flu-vaccination motive study survey suggests that these age groups, particularly those under 30 years, were less interested in the question. Therefore, both should be included in specific in the education efforts. Since 5.8% of respondents do not want to be vaccinated in the future and 14.8% are undecided, there is potential for persuading these individuals to be vaccinated. Staff with an occupational indication for vaccination were more willing to be re-vaccinated, as the vaccination campaign focuses on ethical responsibility.

#### Reasons for vaccination

The survey found that self-protection and preexisting illness was the dominant motive for vaccination. Therefore, considering the state of knowledge [25], [26], [27] in future annual vaccination campaigns, the protective effect of influenza vaccination will be at the focus of the argumentation on the importance of vaccination not only for self-protection but also for the protection of others. While the WHO lists vaccination being unsafe for pregnant women as one of the five myths against vaccination [17], the preventive effect of flu vaccination during pregnancy could be more specifically addressed in the future, as recommended in Sweden [28], because influenza can have a more severe course in pregnant women.

In the flu-vaccination motive study, almost 1/5 of those vaccinated were vaccinated on doctor’s recommendation. Schmid et al. [29] found that individuals who had not received a direct vaccination recommendation from medical personnel were less frequently vaccinated. Since the occupational medical service at GUH offers vaccination both on its premises and within other clinics and institutes, this approach should definitely be maintained and expanded for trainees and students. Since 18.9% were vaccinated by their general practitioner and 8.9% were motivated by the general practitioner to get vaccinated, it makes sense for the GUH to send a motivational letter to general practices in preparation for the flu vaccination campaign.

#### Reasons for non-vaccination

Regardless of the professional group, the perception of one’s own low risk of infection, followed by doubts about the protective effect and fear of side effects dominated in the flu-vaccination motive study. In an analysis conducted in Poland, the main reasons for non-vaccination were similar, e.g., good health (27.6%) and lack of trust in the effectiveness of the vaccination (16.8%) [30]. Also, in the evaluation of 470 studies from all WHO regions, the perception of a low risk of illness in most risk groups and in the general population was identified as an obstacle to flu vaccination [29]. This underscores the need for careful risk education to differentiate between influenza and other respiratory infections and it is necessary to clarify that although influenza is often survived without permanent health impairments, depending on accompanying health conditions such as asthma, chronic bronchitis, chronic obstructive pulmonary disease, chronic cardiovascular, liver or kidney diseases, diabetes or another metabolic disease, neurological or neuromuscular underlying disease, and compromised immune response [31], permanent damage or even death can occur, and that vaccination significantly reduces both the incidence and severity of the disease. The notable increase in motivation in some groups for vaccination for self-protection and herd immunity was possibly influenced by the awareness of the risk of infection by COVID-19.

The third most important reason against influenza vaccination identified in the flu-vaccination motive study was the risk of possible side effects. This pattern is in line with Schmid et al. [29]. In the annual information campaign on flu vaccination at the GUH, it should therefore be pointed out that the majority of vaccinated individuals tolerate the vaccination without side effects and only a small part react with temporary discomfort from mild flu-like symptoms. It is necessary to counter the myth that the flu vaccination can cause influenza [17], [18].

Regarding the reasons against flu vaccination, there were differences in doubts about the protective effect, low infection risk for one’s self, and fear of side effects. This indicates that education efforts must particularly address these three features. Moreover, the social benefit of vaccination, such as herd immunity and patient protection through vaccinated personnel, should be given high priority, which is also a conclusion of the systematic review by Schmid et al. [29].

## Limitation

A cross-sectional survey is only of limited value in determining the influence of the COVID-19 pandemic: Only longitudinal studies are suitable for in-depth analysis.

Although a relevant selection effect is rather unlikely with a response rate of >30%, a bias cannot be excluded because only those who were open to the question responded. However, the respondents answered all questions so that all questionnaires could be evaluated. A hindsight bias cannot be ruled out for the assessment of the influence of the COVID-19 pandemic on vaccination motivation.

## Conclusions

Because a timely reminder of vaccination was essential for 16.7% of staff, starting with 2024, annually around 17. September (the World Patient Safety Day) all employees, students and trainees at the GUH will get a concise updated information sheet via intranet on the importance of flu vaccination with the option of personal advice from the occupational medical service at the same time the hand-out is announcing the vaccination dates in the clinics and institutes.

As a first step in implementing these measures on September 23, 2024 a hand-out was published on the GUH intranet page and on the company medical service website (see Attachment 2), and at the same time an email was sent to all offices of the clinics and institutes as well as to the nursing service managers with the request that it be forwarded to all employees. This hand-out was also sent to the dean of studies, the vocational school and to the practical instructions and were also distributed throughout the house via the internal mail.

Since a quarter of trainees and students participating in the survey were unvaccinated, the GUH will therefore increasingly cover the protective effect of vaccination and health protection through vaccination, as well as the benefits and risks of vaccination in the microbiology and pulmology lectures for medical students in 9. Semester. 

Following the previous annual campaigns for vaccination by the medical director and the occupational medical service, in the fourth quarter of 2024, UMG Live – the GUH employee magazine - will provide information to all GUH employees from hospital hygiene and the company medical service, in which the effectiveness of the influenza vaccination will be highlighted and employees are reminded about the central appointments of the central vaccination campaign in-house or in the outpatient clinic of the company medical service.

## Notes

### Authors’ ORCID

- Axel Kramer: 0000-0003-4193-2149
- Thomas Kohlmann: 0000-0002-5956-8309

### Ethical approval

The survey was approved by the data protection department and by the General Staff Council of University Medicine Greifswald on February 2, 2022.

### Funding

This research received no external funding.

### Acknowledgement 

We would like to thank Nora Katharina Schmid-Küpke und Julia Neufeind, Robert-Koch Institute, Department 2 Epidemiology and Health Monitoring, Berlin, Germany, for their support in the structuring the manuscript and interpreting the results.

### Competing interests

The authors declare that they have no competing interests.

## Supplementary Material

Questionnaire

Information on Flu Vaccination for Employees, Students, and Trainees of the University Medicine Greifswald

## Figures and Tables

**Table 1:**
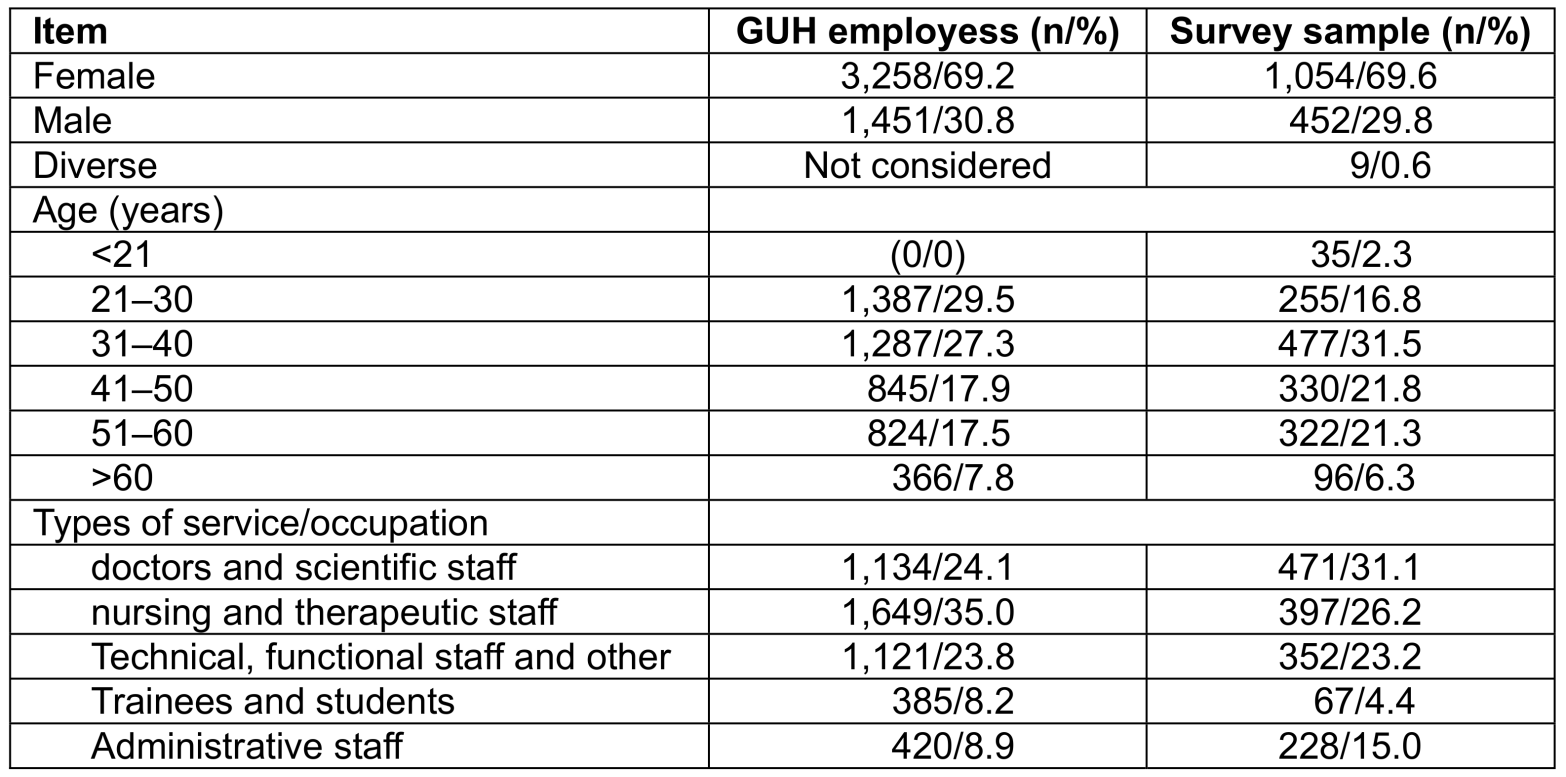
Baseline data of participants in the survey and of all GUH employees

**Table 2:**
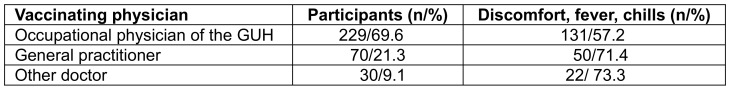
Vaccinating physicians and frequency of reported side effects

**Table 3:**
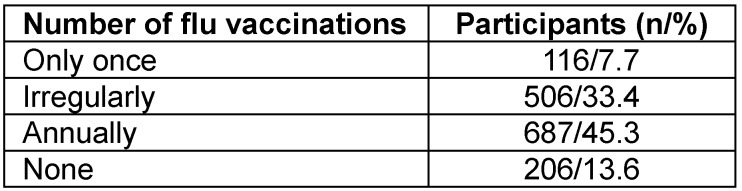
Previous uptake of flu vaccinations

**Table 4:**
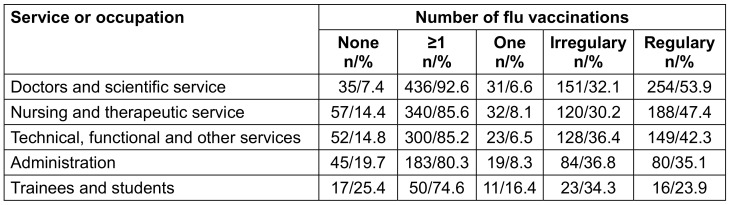
Number of flu vaccinations depending on type of service or occupation

**Table 5:**
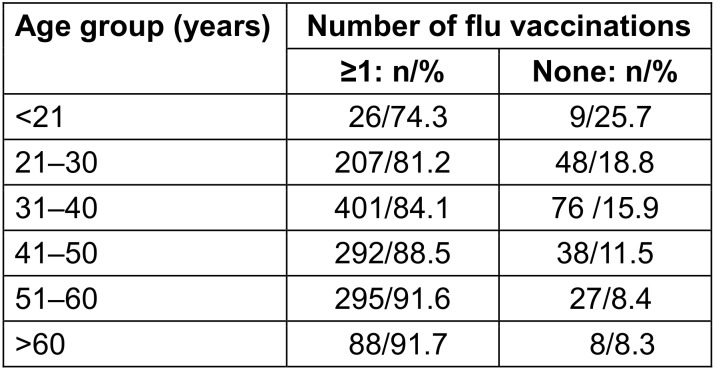
Number of flu vaccinations depending on age

**Table 6:**
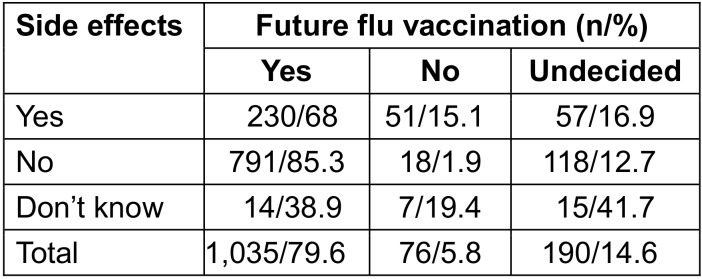
Willingness for future vaccinations depending on the occurrence of side effects

**Table 7:**
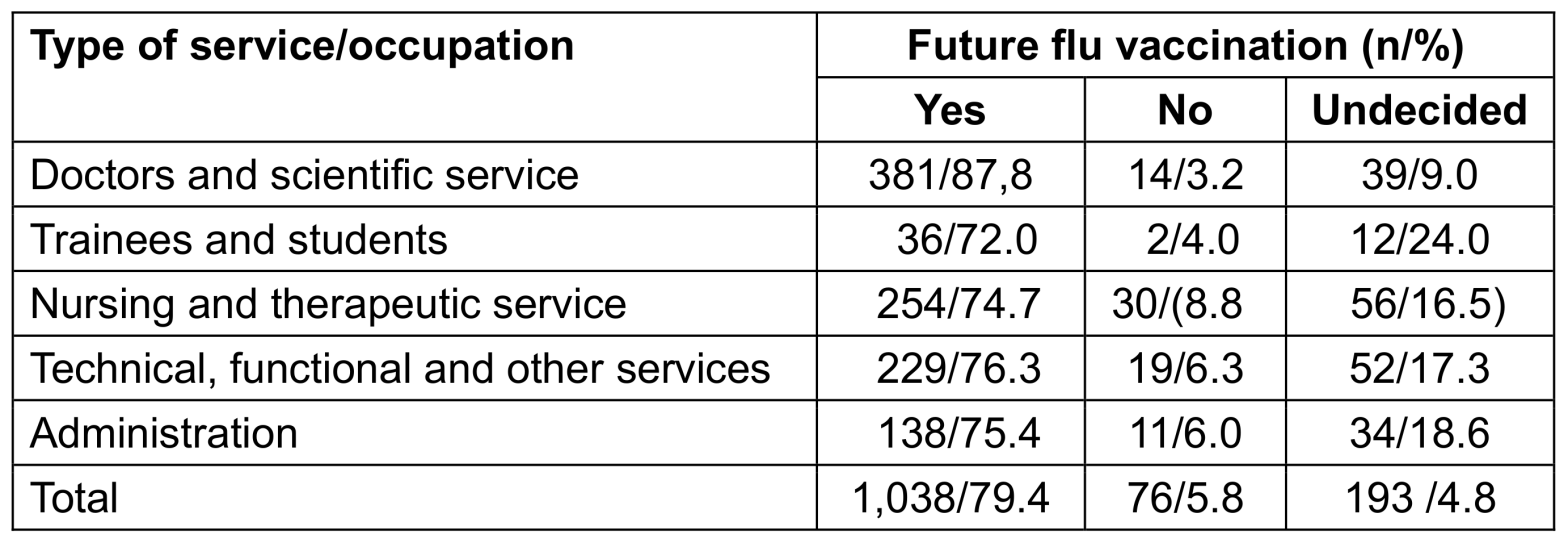
Future willingness to receive flu vaccination among vaccinated respondents by type of service/occupation

**Table 8:**
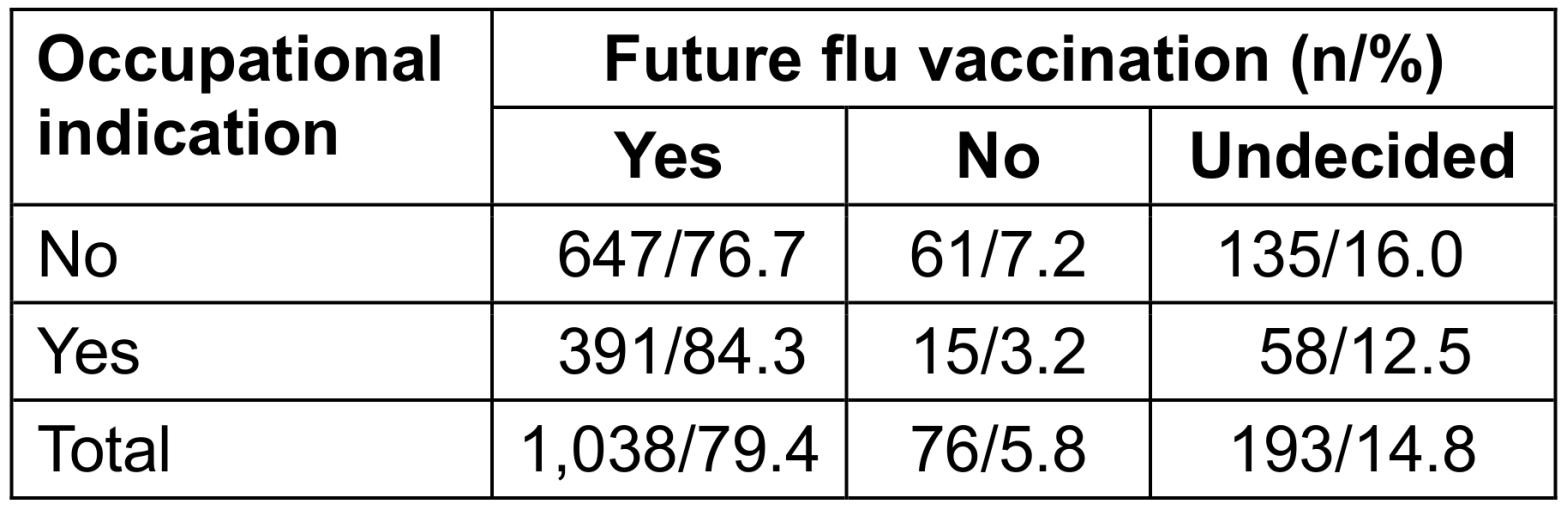
Willingness for future flu vaccinations depending on occupational indication

**Table 9:**
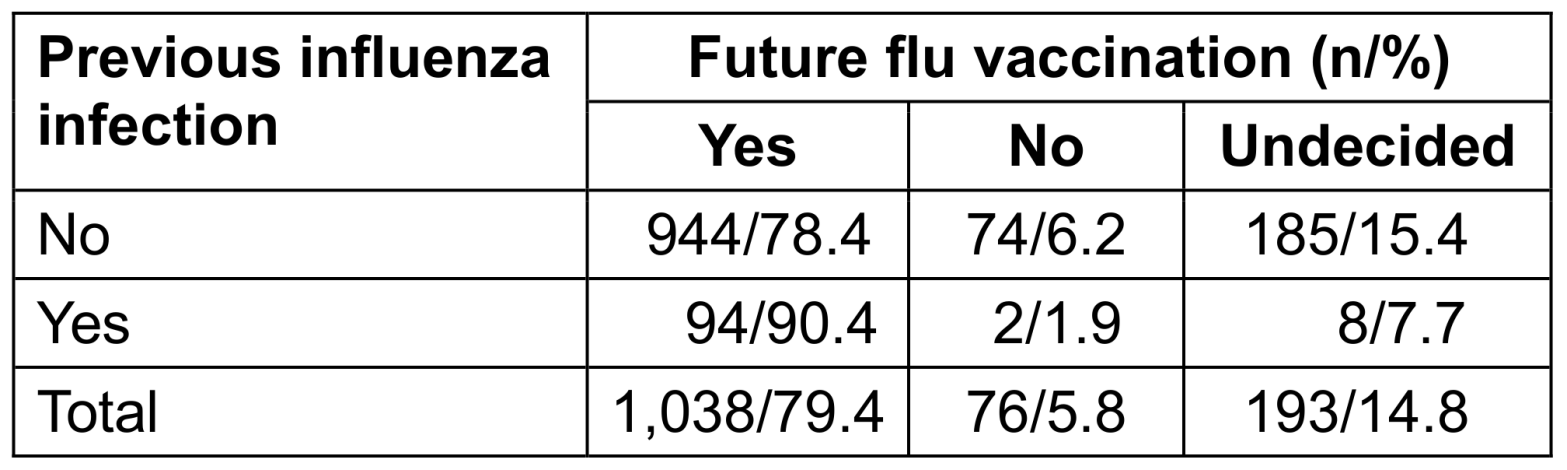
Readiness for future flu vaccinations depending on previous influenza infection

**Table 10:**
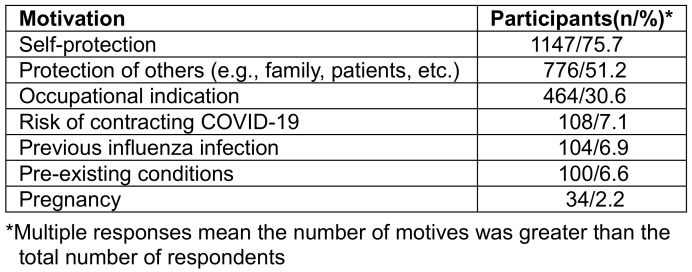
Motivation for flu vaccination uptake among participants who had been vaccinated at least once before

**Table 11:**
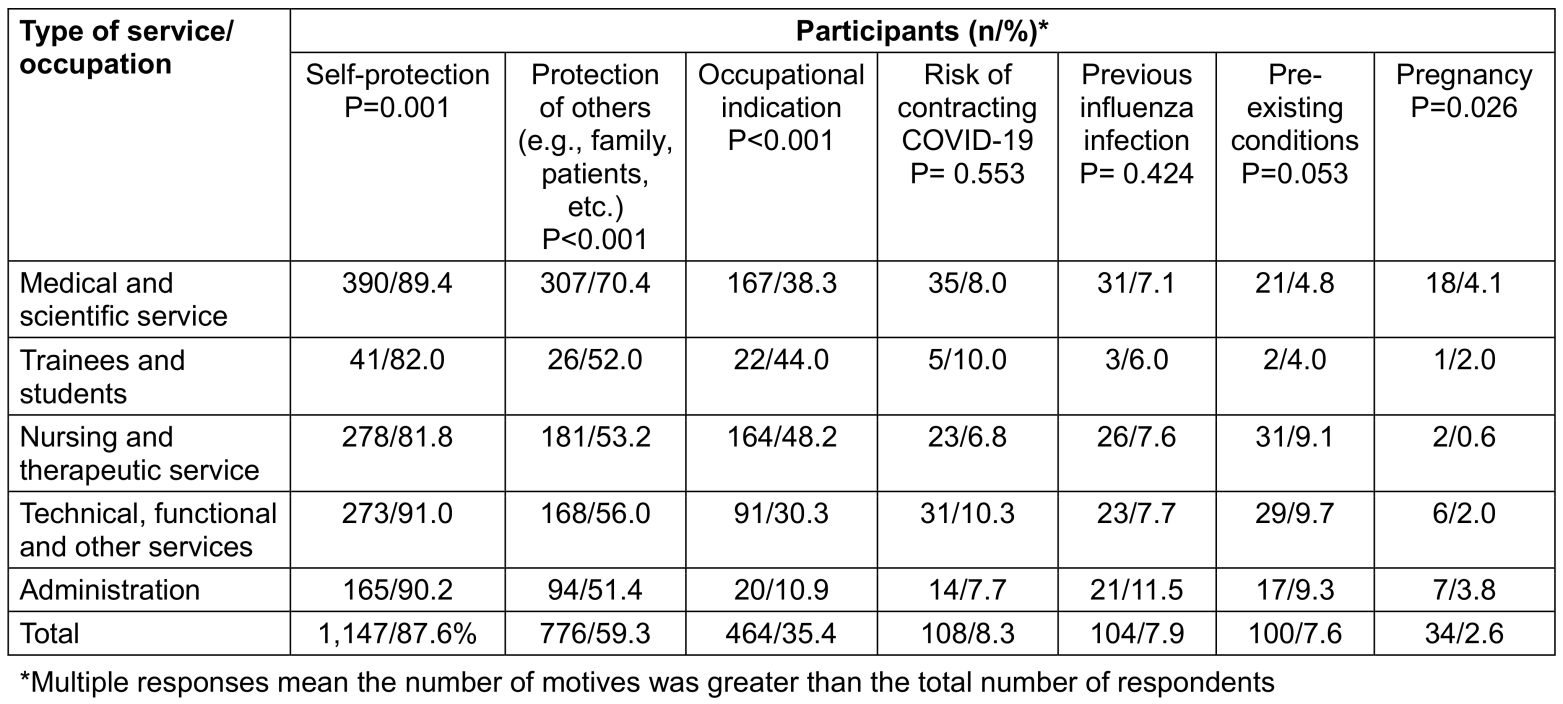
Motivation for flu vaccination uptake among participants who had been vaccinated at least once before, stratified by occupation

**Table 12:**
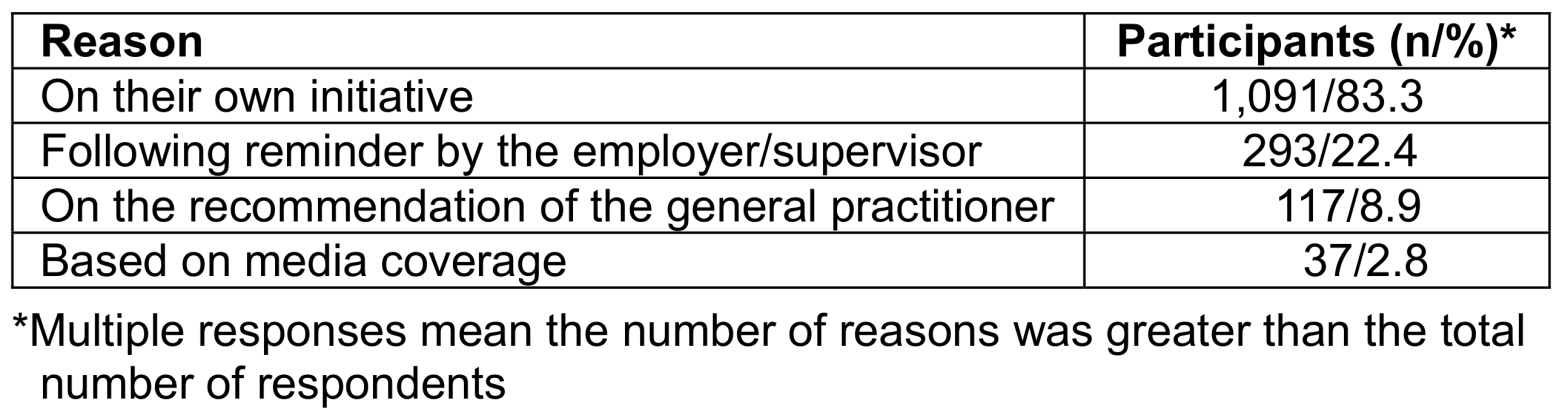
Reasons for vaccination

**Table 13:**
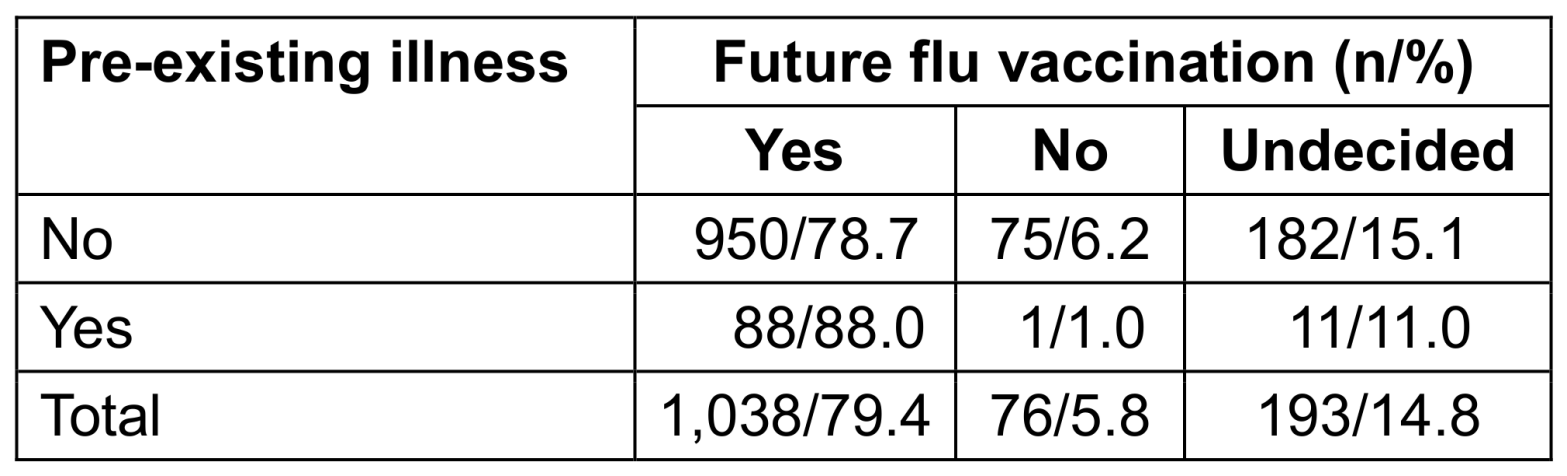
Readiness for future flu vaccinations depending on pre-existing illness

**Table 14:**
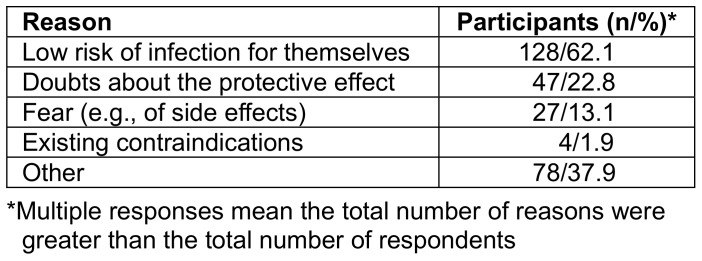
Reasons for non-vaccination

**Table 15:**
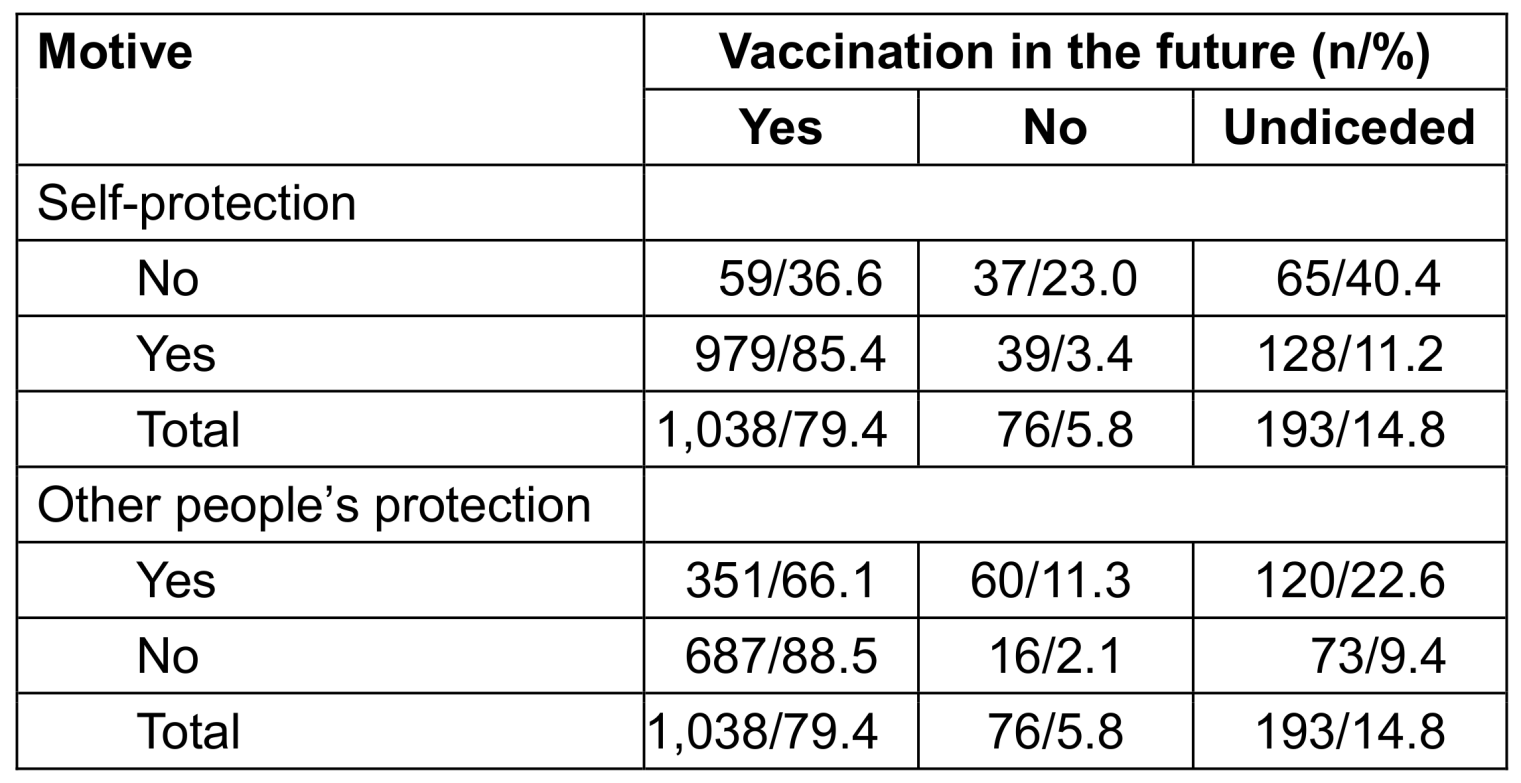
Willingness for future vaccinations depending on the vaccination motivation

**Table 16:**
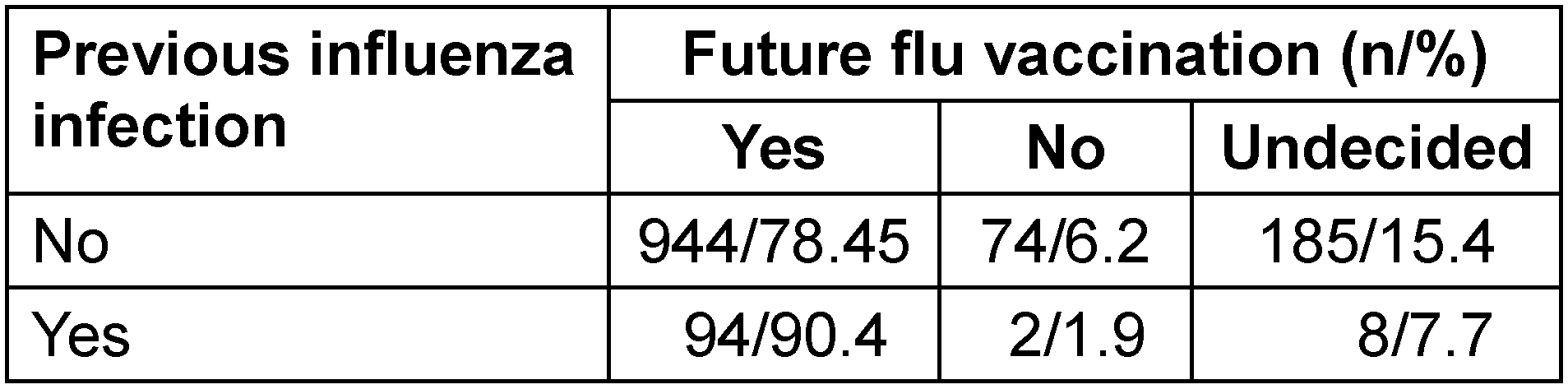
Willingness for future flu vaccinations depending on previous influenza infection

**Table 17:**
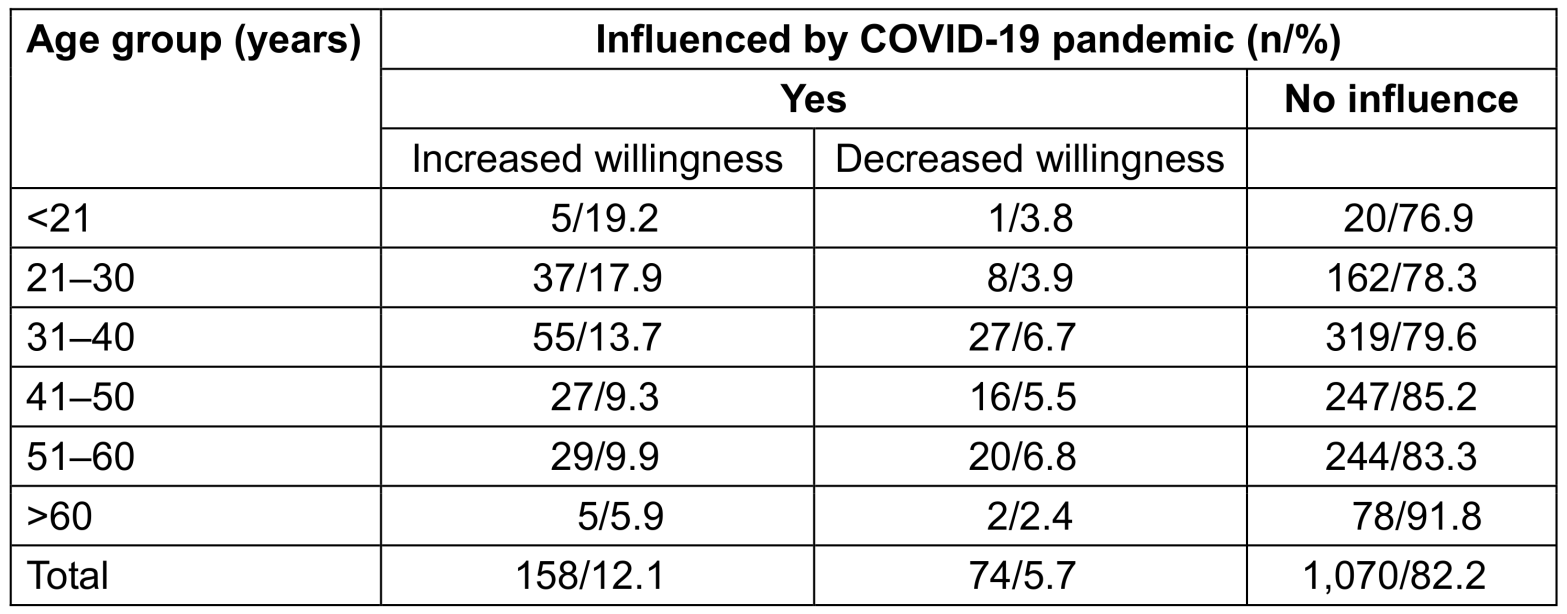
Age-dependent influence on flu vaccination by the COVID-19 pandemic among vaccinated respondents

**Table 18:**
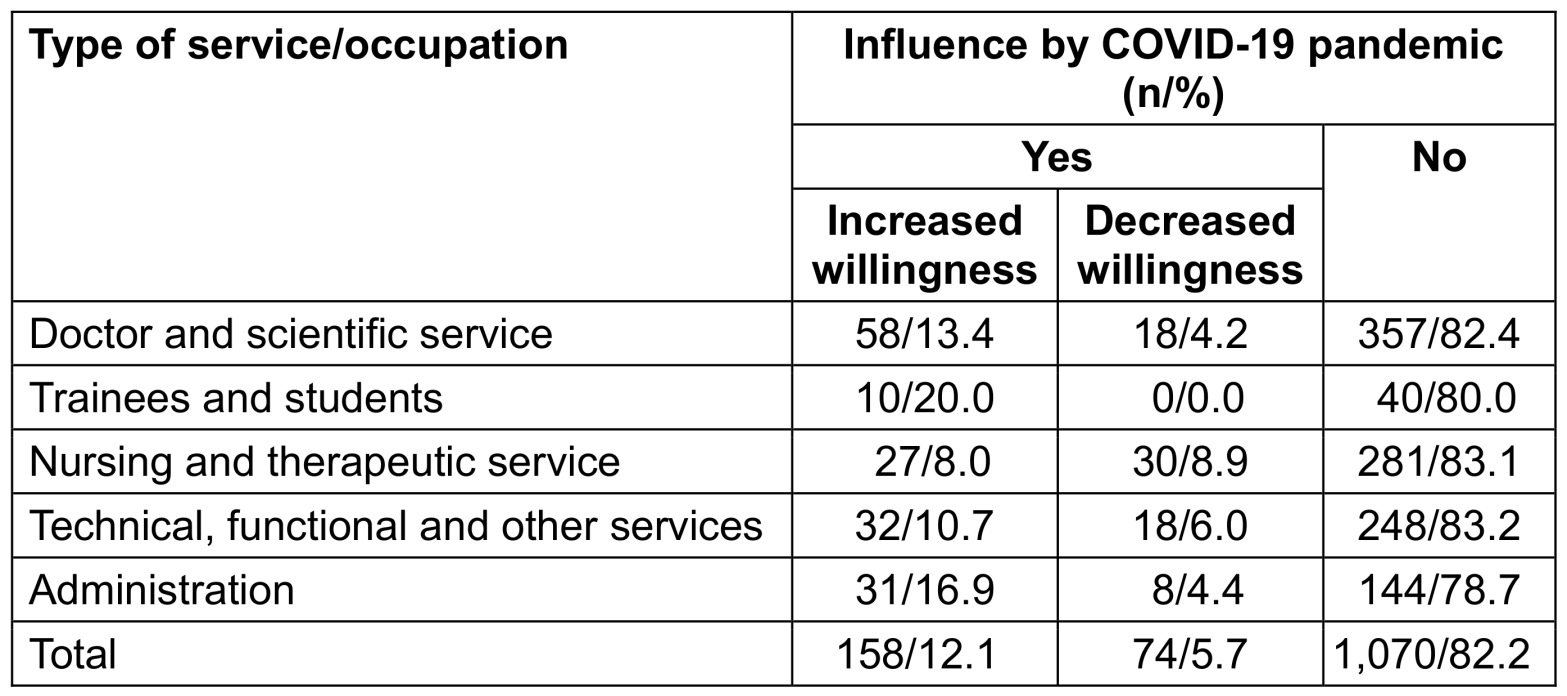
Influence of the Covid-19 pandemic on influenza vaccination among vaccinated respondents by type of service/occupation
