# Supplementary material for: Online survey on barriers and drivers to flu vaccination among staff at a German university hospital during the Covid-19 pandemic 2022 (flu-vaccination motive study)
Source: GMS Hyg Infect Control. 2024 Dec 16;19:Doc64. doi: 10.3205/dgkh000519 (PMC11730181; doi:10.3205/dgkh000519)
Supplement: Questionnaire [file HIC-19-64-s-001.pdf]

Attachment

Questionnaire on the motivation for flu vaccination uptake by employees at University Medicine Greifswald

As part of the scientific evaluation of flu vaccination at University Medicine Greifswald, we appeal to you for your support. Answering the few questions using the checkbox method without free text answers will take no more than one minute.
Explanation: In the context of the COVID-19 pandemic, it is especially important to prevent seasonal influenza. We would be grateful if you would complete the questionnaire to analyze your motives for receiving the flu vaccination. Anonymity is guaranteed thanks to the electronic response.

General information

1.1 Your department

Operating division (OD) Purchasing ☐, OD Finance ☐, OD Commercial Executive Board ☐, OD Medical Executive Board ☐, Dermatology Clinic ☐, Ear, nose and throat clinic ☐,
Hospice ☐, Human Genetics ☐, Hygiene/Environmental Medicine ☐, Immunology ☐, Internal Medicine ☐, Pediatric Surgery ☐, Pediatric Clinic ☐, Clinical Chemistry ☐, Medical Biochemistry ☐, Medical Microbiology ☐, Medical Psychology ☐,Neurosurgery ☐, Neurology ☐, Orthopedics ☐, Pathology ☐, Pathophysiology ☐, Pharmacology ☐, Physiology ☐, Physiotherapy ☐, Pool staff ☐, Psychiatry ☐, Radiology ☐, Forensic Medicine ☐, Student ☐, Transfusion Medicine ☐, Technology ☐, Urology ☐, Laboratory Animal Science ☐, Central OR ☐, Nursing ☐, Federal Volunteer Service ☐, Central Emergency Room ☐, Dentistry, Oral and Maxillofacial Surgery ☐, Dialysis ☐

1.2 Job

Medical service ☐, Scientific service ☐, Administration ☐, Nursing service ☐, Technical service ☐, Trainees ☐, Therapeutic service ☐, Functional service ☐, Student assistant ☐, other ☐

1.3 Age

☐ <20 years
☐ 21- 30 years
☐31- 40 years
☐ 41 – 50 years
☐ 51- 60 years
☐ > 60 years

1.4 Gender

☐ Male
☐ Female
☐ Diverse

2. Questions about your experiences with flu vaccination

2.1. How many influenza vaccinations have you received in your life?
☐ None
☐ One
☐ Two or more
☐ Annually

2.2. For what reasons have you not been vaccinated against influenza so far? (Filter question: Only if the answer to 2.2 is 'None', then end the questionnaire; multiple answers possible)
☐ Doubts about the protective effect
☐ Fear (e.g., of side effects)
☐ Low personal risk of infection
☐ Existing contraindications
☐ Other
End of the questionnaire only if no influenza vaccination has been administered so far.

2.3 Have you experienced any side effects from the flu vaccination in the past?
☐ Yes
☐ No
☐ Don't know

2.4 What side effects did you notice? (Filter question, if 2.3 answered with Yes; multiple answers possible):
☐ Local reactions such as pain, redness, swelling
☐ Temporary malaise, mild fever, chills, or increased sweating
☐ Fatigue
☐ Headaches
☐ Muscle or joint pain
☐ Other

2.5 Has the Covid-19 pandemic influenced your decision regarding the flu vaccination?
☐ No
☐ Yes

2.6 In what way has the pandemic influenced your decision regarding the flu vaccination? (Filter question, if 2.5 answered with Yes. Multiple answers possible)
☐ Increased willingness to be vaccinated (to prevent influenza)
☐ Reduced willingness to be vaccinated (e.g., skepticism about the necessity of the influenza vaccination)

2.7 Please select your motivation for getting the flu vaccination (multiple answers possible):
☐ Self-protection
☐ Protection of others (e.g., family, patients, etc.)
☐ Previous influenza infection
☐ Occupational reason
☐ Pregnancy
☐ Risk of COVID-19 infection
☐ Pre-existing conditions

2.8 What was the reason for your decision to get the flu vaccination? (Multiple answers possible):
☐ On my own initiative
☐ Following a reminder by my employer/supervisor
☐ On the recommendation of the my general practitioner
☐ Due to public reporting in the press, radio and television

2.9 Who vaccinated you?
☐ The occupational health service at UMG
☐ A general practitioner
☐ Another doctor (gynecologist, public health office, etc.)

2.10 Are you satisfied with the effectiveness of the flu vaccination?
☐ Yes
☐ No
☐ Cannot judge

2.11 Will you continue to get vaccinated against influenza?
☐ Yes
☐ No
☐ Undecided

Thank you for your participation!
