# Supplementary material for: Online survey on barriers and drivers to flu vaccination among staff at a German university hospital during the Covid-19 pandemic 2022 (flu-vaccination motive study)
Source: GMS Hyg Infect Control. 2024 Dec 16;19:Doc64. doi: 10.3205/dgkh000519 (PMC11730181; doi:10.3205/dgkh000519)
Supplement: Information on Flu Vaccination for Employees, Students, and Trainees of the University Medicine Greifswald [file HIC-19-64-s-002.pdf]

Information on Flu Vaccination for Employees, Students, and Trainees of the University Medicine Greifswald

In the online survey conducted in 2020 by the Robert Koch Institute in Berlin on flu vaccination, 131 clinics across Germany participated. While the average vaccination rate was 52.9%, it was significantly higher at 75% in the University Medicine Greifswald (UMG). One reason is that vaccinations were offered and explained to employees in their workplace. However, results from UMG's internal online survey in 2022 showed that 5.8% of participants were unwilling to receive future vaccinations, and 58% were undecided. Common reasons for refusal or hesitation included a perceived low personal risk of infection, doubts about the vaccine's effectiveness, and fear of side effects. The WHO (2022) addresses five common myths circulating in the population about influenza vaccination with the following arguments:

Myth 1: "Influenza is not a severe illness, so vaccination is unnecessary." In reality, more than 650,000 people die annually from respiratory manifestations alone. Even though many people recover within a few weeks, particularly individuals with immunosuppression and underlying diseases are at risk of permanent damage, especially to the heart muscle, lungs, brain, kidneys, and ENT areas.

Myth 2: "The influenza vaccine can cause the flu." The flu vaccine contains inactivated virus that cannot cause influenza. If one feels pain or mild fever, it is a normal reaction of the immune system to the vaccine and typically lasts only a day or two.

Myth 3: "The flu vaccine can cause severe side effects." The flu vaccine is proven to be safe. Serious side effects are extremely rare. One in a million people may develop Guillain-Barré Syndrome, which causes muscle weakness and paralysis.

Myth 4: "I got vaccinated and still got the flu; therefore, the vaccine is not effective." As various flu viruses are always circulating, it is possible to contract the flu despite vaccination, since the vaccine only protects against the virus types and subtypes used in it. Generally, vaccination increases the likelihood of being protected against the flu by 40% (CDC 2023) and reduces mortality by 31-83% (Thompson et al. 2018). A vaccination rate of 77% was associated with halving the rate of illness in healthcare staff and reducing infection pressure on the patients cared for.

Myth 5: "I am pregnant and should not get the flu vaccine." Pregnant women should particularly be vaccinated against the flu as their immune system is weaker than usual. The inactivated flu vaccine is safe at any stage of pregnancy.

The University Medicine Greifswald therefore recommends all employees, students, and trainees to take advantage of the free ias well as to protect patients from flu infection by interrupting virus transmission (known as herd immunity).

We recommend getting the flu vaccination at the occupational health service, where fewer side effects have been reported (Flu-Motiv Study 2024). The occupational health service is always available to advise on the health benefits of the influenza vaccination.

Attachment 1 to: Schmidt-Bandelin A, Kohlmann T, Ruback A, Reuter U, Kramer A. Online survey on barriers and drivers to flu vaccination among staff at a German university hospital during the Covid-19 pandemic 2022 (flu-vaccination motive study). GMS Hyg Infect Control. 2024;19:Doc64. DOI: 10.3205/dgkh000519
